# Supplementary material for: Characterizing cell-type spatial relationships across length scales in spatially resolved omics data
Source: Nat Commun. 2025 Jan 3;16:350. doi: 10.1038/s41467-024-55700-1 (PMC11699133; doi:10.1038/s41467-024-55700-1)
Supplement: Supplementary file 1 — Supplementary Information for Characterizing cell-type spatial relationships across length scales in spatially resolved omics data [file 41467_2024_55700_MOESM1_ESM.pdf]

Supplementary Information for

Characterizing cell-type spatial relationships across length scales in spatially resolved omics data

Rafael dos Santos Peixoto[1,2], Brendan F. Miller[1,2], Maigan A. Brusko[3], Gohta Aihara[1,2], Lyla Atta[1,2], Manjari Anant[1,4], Mark A. Atkinson[3], Todd M. Brusko[3], Clive H. Wasserfall[3], Jean Fan[*,1,2]

[1] Center for Computational Biology, Whiting School of Engineering, Johns Hopkins University, Baltimore, MD 21211

[2] Department of Biomedical Engineering, Johns Hopkins University, Baltimore, MD 21218,

[3] Department of Pathology, Immunology, and Laboratory Medicine, University of Florida, Gainesville, FL 32610

[4] Department of Neuroscience, Johns Hopkins University, Baltimore, MD 21205

* Correspondence should be addressed to:

Jean Fan (jeanfan@jhu.edu)

Supplementary Figures

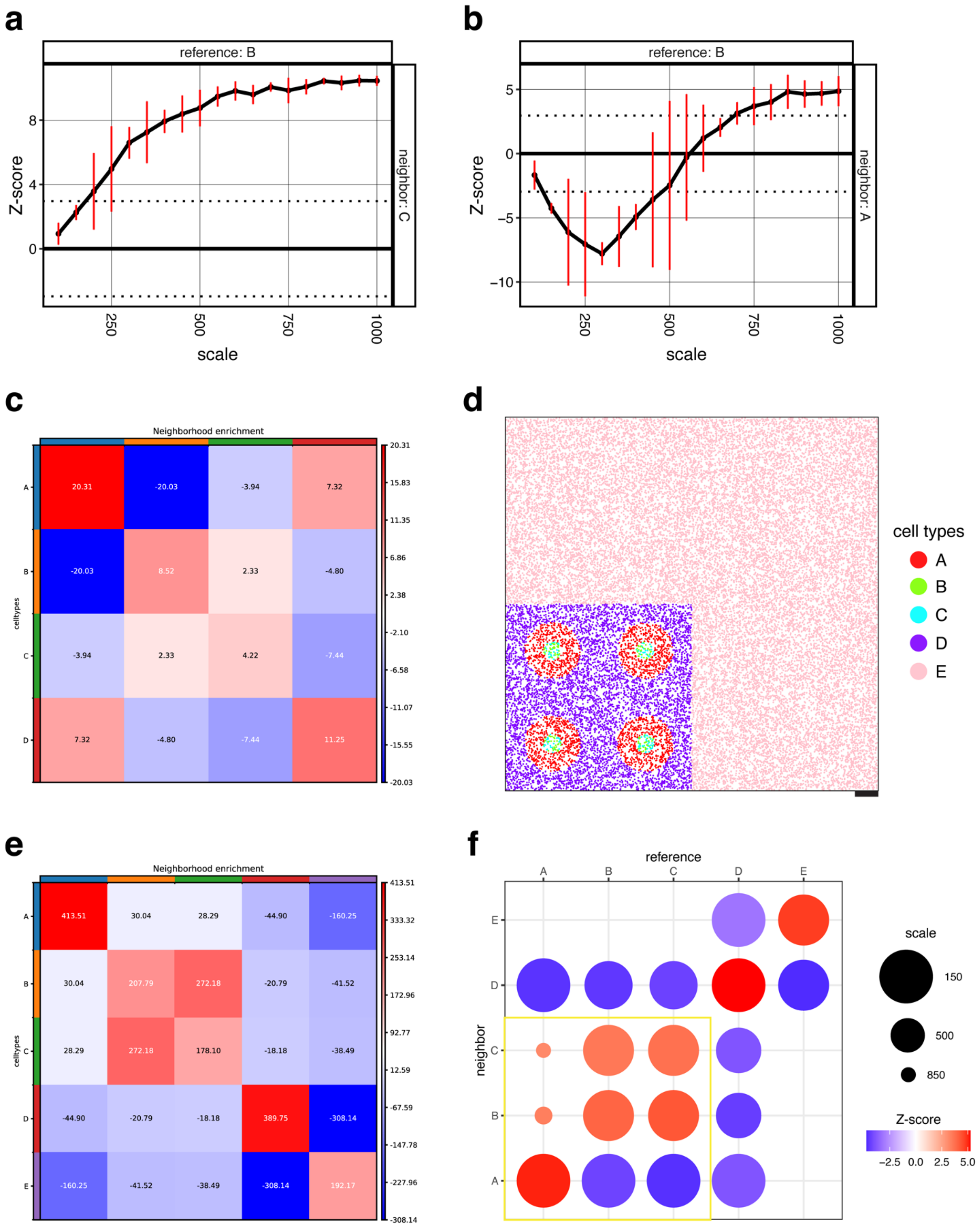

Supplementary Figure 1. CRAWDAD and Squidpy's neighborhood enrichment implementation on simulated data. CRAWDAD's multi-scale spatial relationship trend plot for **a.** reference cell-type B and neighbor cell-type C and **b.** for reference cell-type B and neighbor cell-type A. The horizontal black dotted lines represent the Z-score significance threshold corrected for multiple testing (Z-score = ±2.96). The vertical red bars represent the error bars of ± one standard deviation from the mean Z-score estimated using permutations. **c.** Squidpy's neighborhood enrichment analysis on the simulated data. **d**. New simulated data that included cell-type E contouring the previous original dataset. **e.** Squidpy's neighborhood enrichment analysis on the new simulated data in (d) shows changing cell-type relationships compared to (c). **f.** Summary visualization of CRAWDAD's multi-scale cell-type spatial relationship analysis of the new simulated data. The yellow box highlights that the cell-type relationships found in the analysis of the original simulated data were maintained.

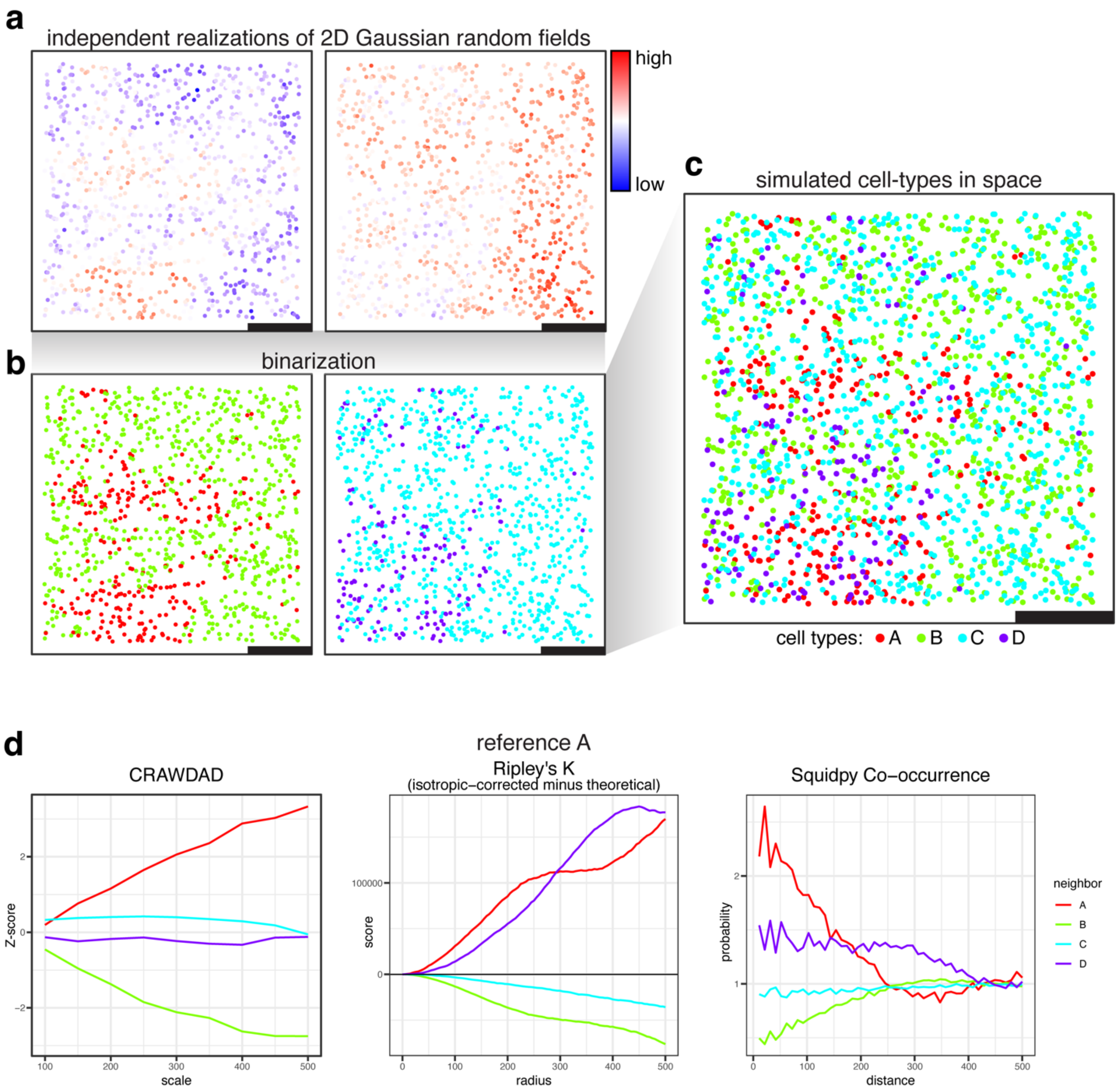

Supplementary Figure 2. Creation of the simulated dataset. **a**. Two independent Gaussian random fields with uniformly sampled spatial positions representing 1000 cells. **b**. These spatial positions are assigned a cell type based on the random field's value. **c**. Both fields are combined to generate one dataset with 4 cell types. **d**. Relationships trends obtained by each method using cell type A as the reference cell type.

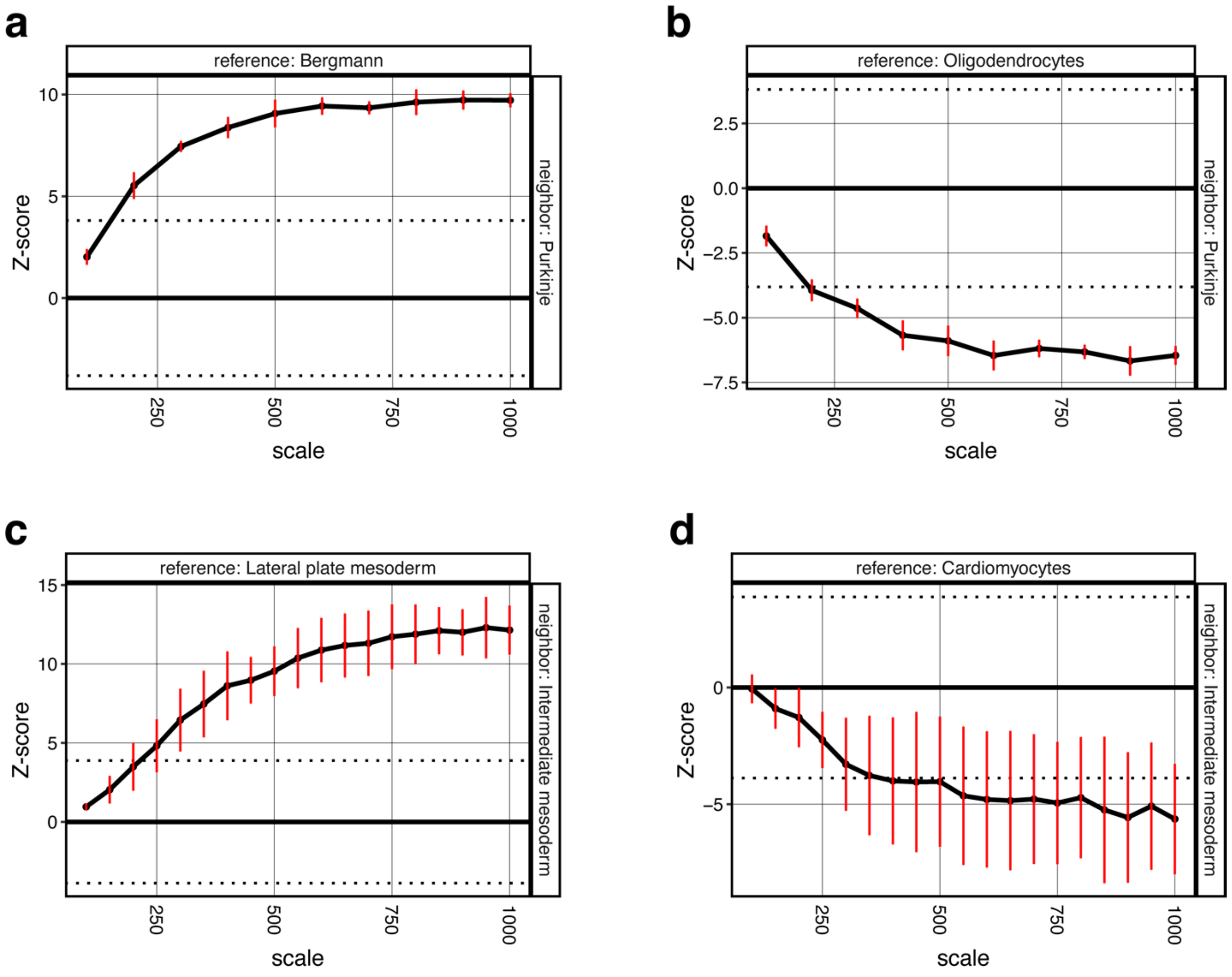

Supplementary Figure 3. Complementary CRAWDAD analysis of the mouse cerebellum and mouse embryo datasets. **a-d**. The multi-scale spatial relationship trend plot for (**a**) reference cell-type Bergmann glia and neighbor cell-type Purkinje neurons; (**b**) reference cell-type Oligodendrocytes and neighbor cell-type Purkinje neurons; (**c**) reference cell-type lateral plate mesoderm and neighbor cell-type intermediate mesoderm; (**d**) reference cell-type cardiomyocyte and neighbor cell-type intermediate mesoderm. The horizontal black dotted lines represent the Z-score significance threshold corrected for multiple testing (Z-score = ±3.81 for a-b and Z-score = ±3.88 for c-d). The vertical red bars represent the error bars of ± one standard deviation from the mean Z-score estimated using permutations.

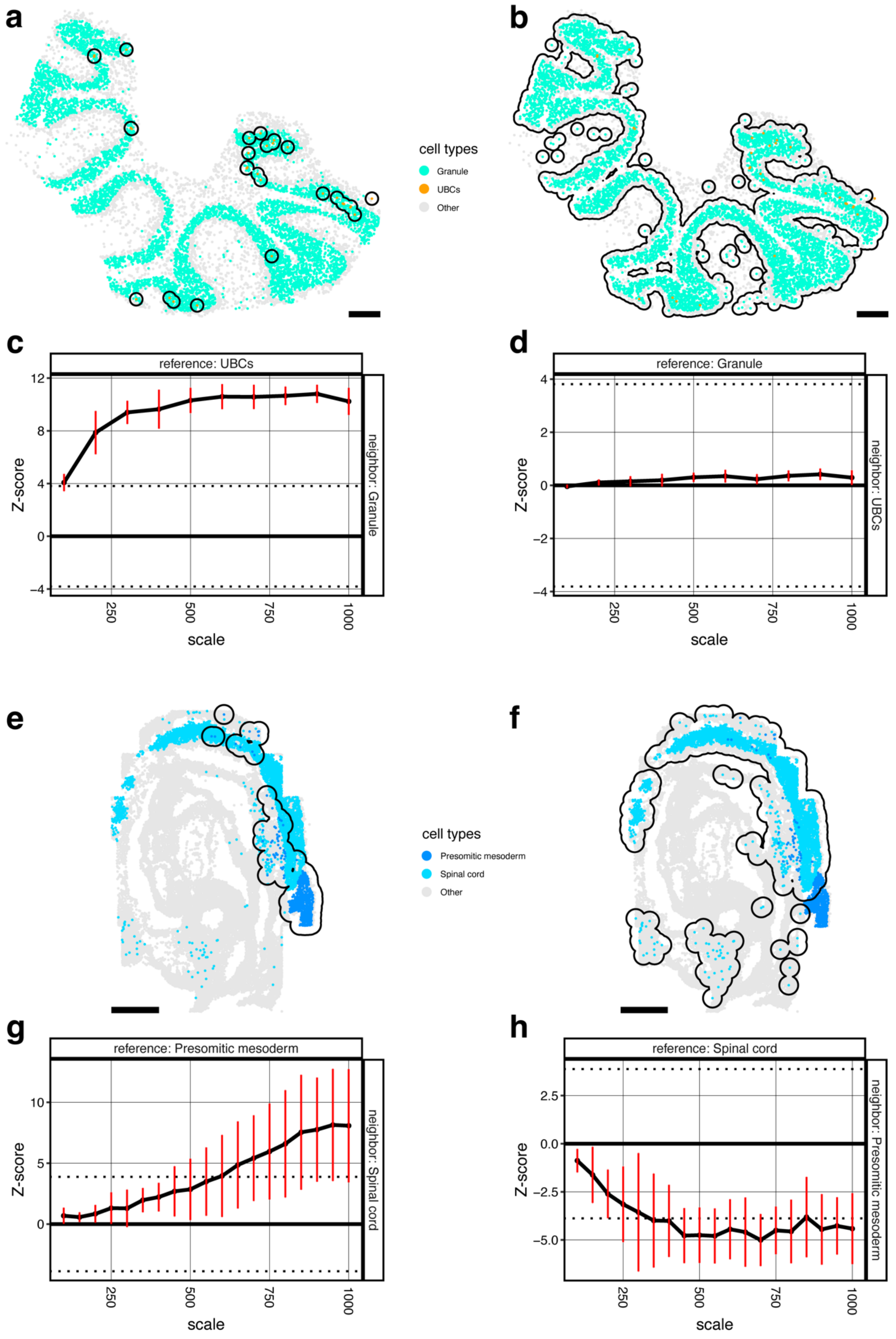

a
b
cell types
Granule
UBCs
Other
c
d
reference: UBCs
neighbor: Granule
Z-score
scale
reference: Granule
neighbor: UBCs
e
f
cell types
Presomitic mesoderm
Spinal cord
Other
g
h
reference: Presomitic mesoderm
neighbor: Spinal cord
reference: Spinal cord
neighbor: Presomitic mesoderm

Supplementary Figure 4. Sample asymmetric cell-type spatial relationships. Spatial visualization of cells in the cerebellum with the neighborhood of UBCs (**a**) and Granule cells (**b**) outlined. CRAWDAD's multi-scale spatial relationship trend plot for UBCs and Granule cells with UBCs as the reference cell type and Granule cells as the neighboring cell type (**c**) and vice versa (**d**). Spatial visualization of cells in the embryo with the neighborhood of presomitic mesoderm cells (**e**) and spinal cord cells (**f**) outlined. CRAWDAD's multi-scale spatial relationship trend plot for presomitic mesoderm cells and spinal cord cells with presomitic mesoderm cells as the reference cell type and spinal cord cells as the neighboring cell type (**g**) and vice versa (**h**). Scale bars correspond to 250μm. The horizontal black dotted lines represent the Z-score significance threshold corrected for multiple testing (Z-score = ±3.81 for c-d and Z-score = ±3.88 for g-h). The vertical red bars represent the error bars of ± one standard deviation from the mean Z-score estimated using permutations.

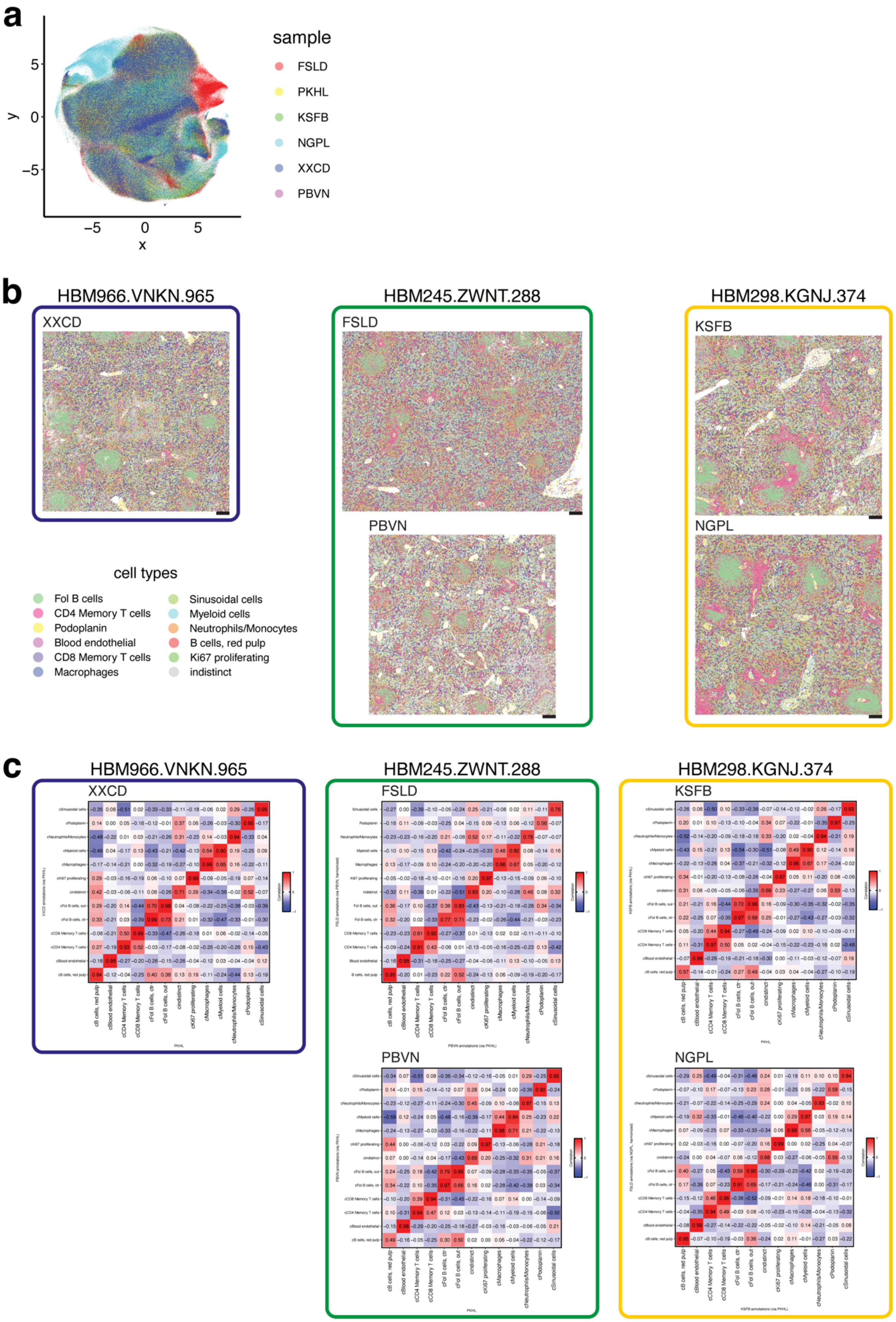
a
sample
FSLD
PKHL
KSFB
NGPL
XXCD
PBVN
y
x
5
0
−5
−5
0
5
b
HBM966.VNKN.965
XXCD
HBM245.ZWNT.288
FSLD
PBVN
HBM298.KGNJ.374
KSFB
NGPL
cell types
Fol B cells
CD4 Memory T cells
Podoplanin
Blood endothelial
CD8 Memory T cells
Macrophages
Sinusoidal cells
Myeloid cells
Neutrophils/Monocytes
B cells, red pulp
Ki67 proliferating
indistinct
c
HBM966.VNKN.965
XXCD
HBM245.ZWNT.288
FSLD
PBVN
HBM298.KGNJ.374
KSFB
NGPL

Supplementary Figure 5. CODEX Human Spleen Samples. **a.** Reduced-dimension visualization of the samples. **b.** Spatial visualization of cell types in the different samples and patients. **c.** Heatmaps visualizing transcriptional correlations between label-transferred cell types in spleen samples and the originally annotated cell types in reference spleen sample PKHL. Scale bars correspond to 250µm.

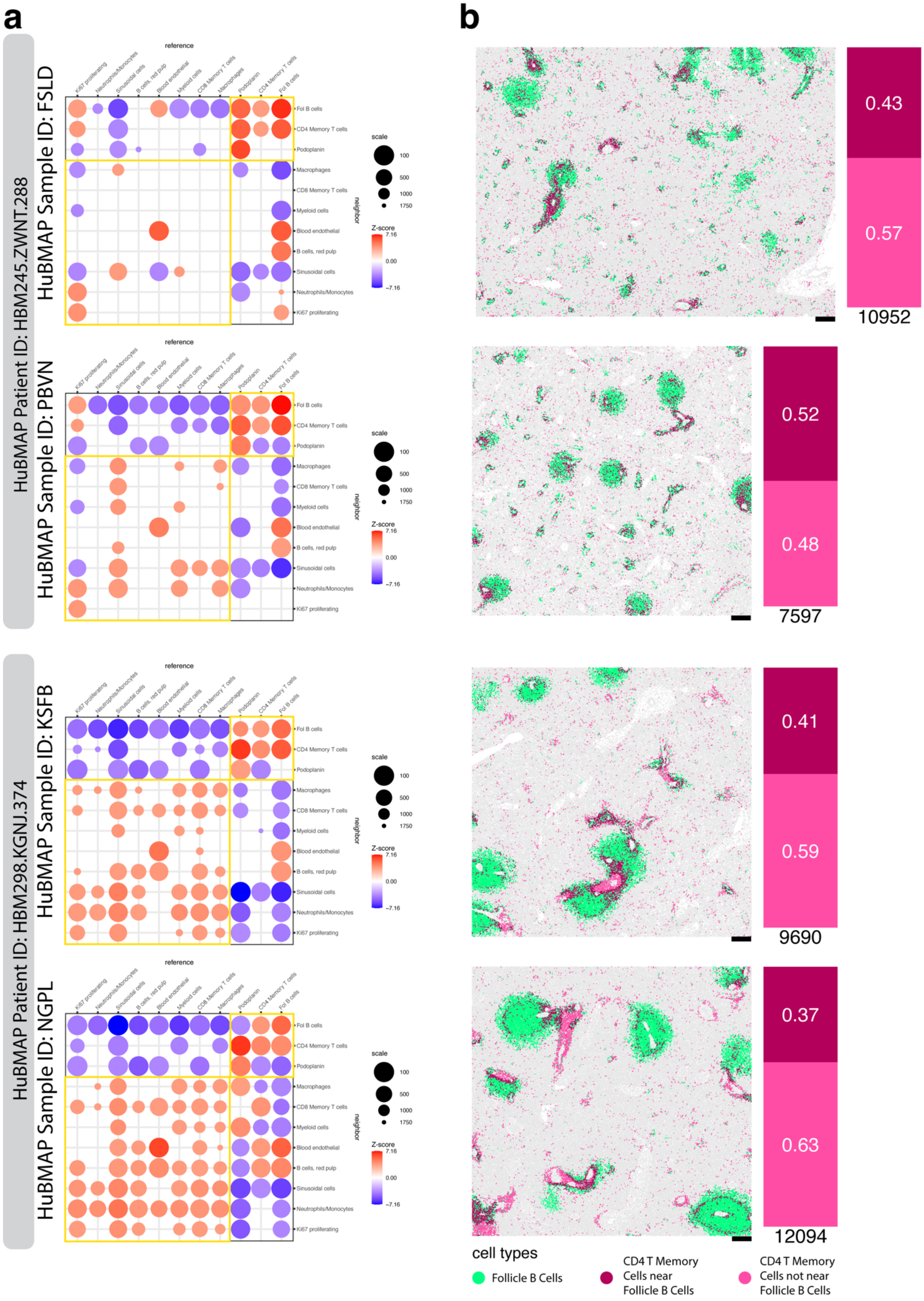
a
b
HuBMAP Patient ID: HBM245.ZWNT.288
HuBMAP Sample ID: FSLD
HuBMAP Sample ID: PBVN
HuBMAP Patient ID: HBM298.KGNJ.374
HuBMAP Sample ID: KSFB
HuBMAP Sample ID: NGPL
reference
neighbor
Ki67 proliferating
Neutrophils/Monocytes
Sinusoidal cells
B cells, red pulp
Blood endothelial
Myeloid cells
CD8 Memory T cells
Macrophages
Podoplanin
CD4 Memory T cells
Fol B cells
scale
100
500
1000
1750
Z-score
7.16
0.00
−7.16
0.43
0.57
10952
0.52
0.48
7597
0.41
0.59
9690
0.37
0.63
12094
cell types
Follicle B Cells
CD4 T Memory Cells near Follicle B Cells
CD4 T Memory Cells not near Follicle B Cells

Supplementary Figure 6. Analysis of other spleen samples. **a.** Summary visualization of the multi-scale cell-type spatial relationship analysis for datasets FSLD and PBVN from patient HBM245.ZWNT.288 and for datasets KSFB and NGPL from patient HBM298.KGNJ.374. **b.** On the left, the spatial visualization of the subsets of CD4+ Memory T cells near and not near Follicle B cells, and Follicle B cells. On the right, the number of CD4+ Memory T cells (n) and the proportion of those that are near Follicle B cells. Scale bars correspond to 250µm.

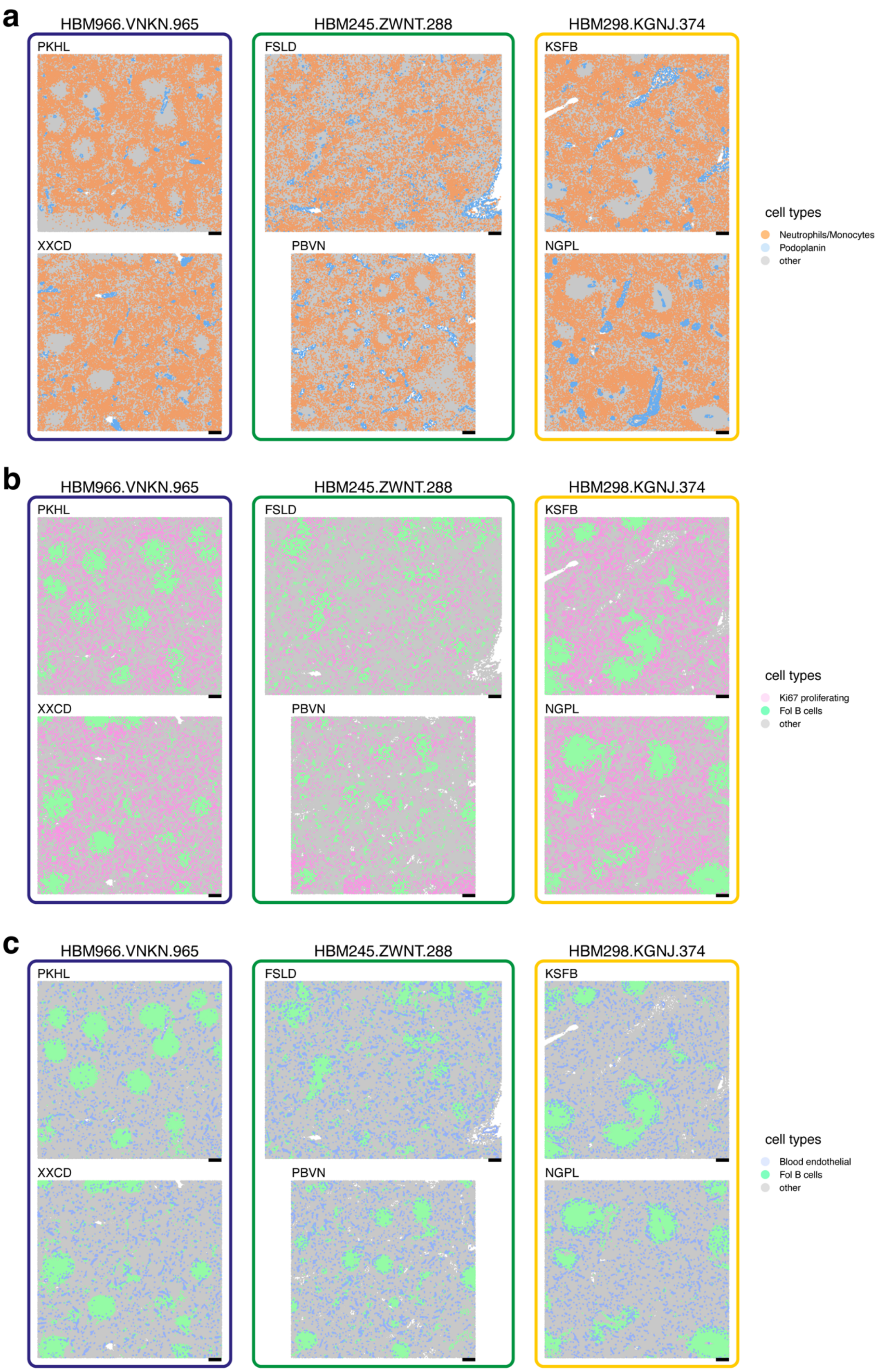

a
HBM966.VNKN.965
HBM245.ZWNT.288
HBM298.KGNJ.374
PKHL
FSLD
KSFB
XXCD
PBVN
NGPL
cell types
Neutrophils/Monocytes
Podoplanin
other
b
HBM966.VNKN.965
HBM245.ZWNT.288
HBM298.KGNJ.374
PKHL
FSLD
KSFB
XXCD
PBVN
NGPL
cell types
Ki67 proliferating
Fol B cells
other
c
HBM966.VNKN.965
HBM245.ZWNT.288
HBM298.KGNJ.374
PKHL
FSLD
KSFB
XXCD
PBVN
NGPL
cell types
Blood endothelial
Fol B cells
other

Supplementary Figure 7. Spatial visualization of selected cell types in the spleen samples. **a.** Visualization of Neutrophils/Monocytes and Podoplanin-enriched cells, exemplifying cell-type spatial relationship patterns consistent across all samples. **b.** Visualization of Ki67 and Follicular B cells, exemplifying cell-type spatial relationship patterns consistent across patients. **c.** Visualization of Blood endothelial and Follicular B cells, exemplifying inconsistent cell-type spatial relationship patterns. Scale bars correspond to 250μm.

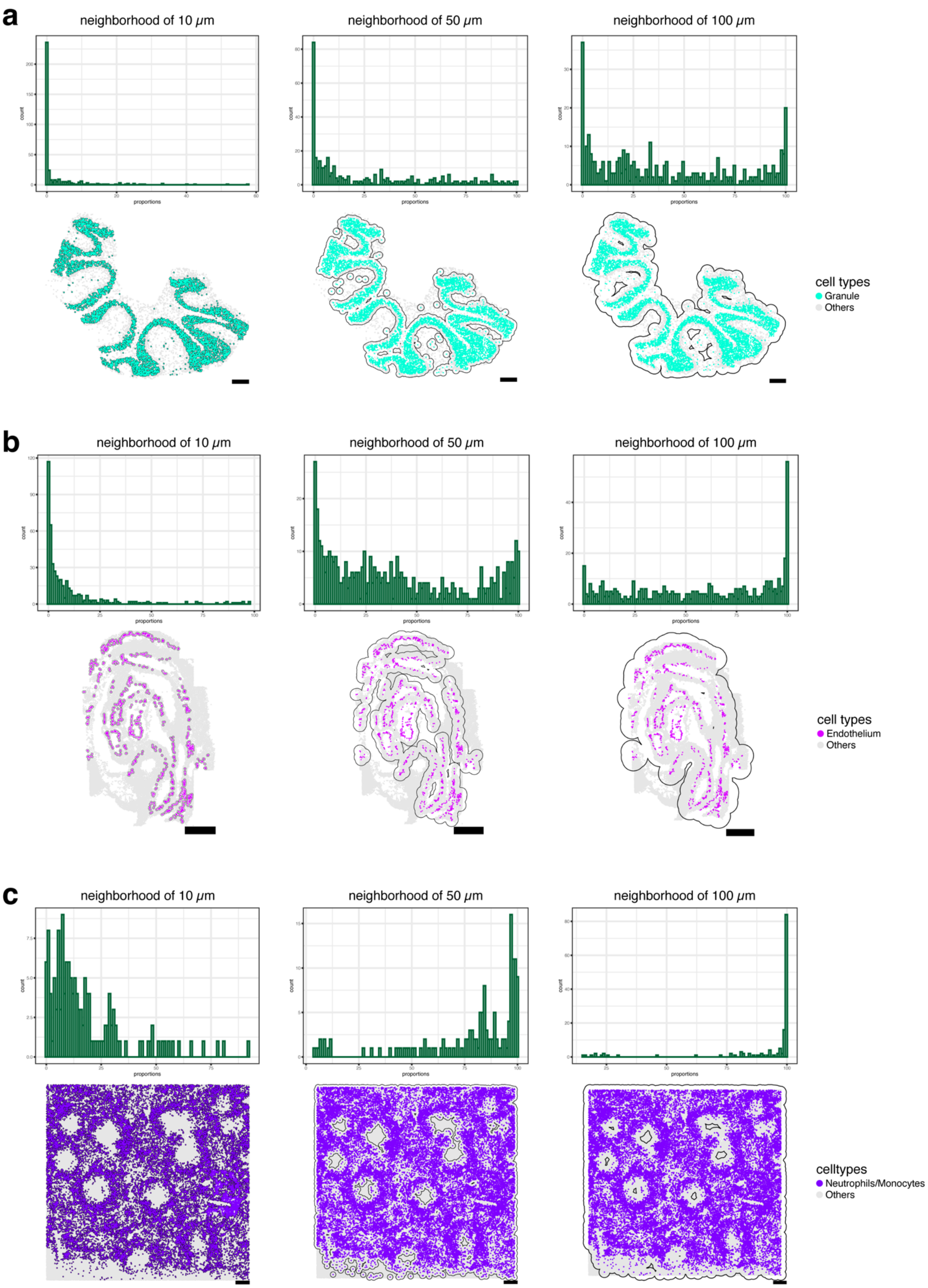

a
neighborhood of 10 μm
neighborhood of 50 μm
neighborhood of 100 μm
count
proportions
cell types
Granule
Others
b
neighborhood of 10 μm
neighborhood of 50 μm
neighborhood of 100 μm
count
proportions
cell types
Endothelium
Others
c
neighborhood of 10 μm
neighborhood of 50 μm
neighborhood of 100 μm
count
proportions
celltypes
Neutrophils/Monocytes
Others

Supplementary Figure 8. The effects of the neighborhood size. **a**-**c**. Histogram of the cell-type proportion of cells from the neighbor of each cell type given neighborhood sizes of 10, 50 and 100 µm (top). Corresponding spatial visualization of the neighborhood as a black outline for the chosen cell type (bottom). (**a**) Visualization of the cerebellum proportions and spatial visualization Granule neighborhoods. (**b**) Visualization of the embryo proportions and spatial visualization of the Endothelium neighborhoods. (**c**) Visualization of the proportions and spatial visualization of the Neutrophils/Monocytes neighborhoods in the spleen sample PKHL. Scale bars correspond to 250µm.

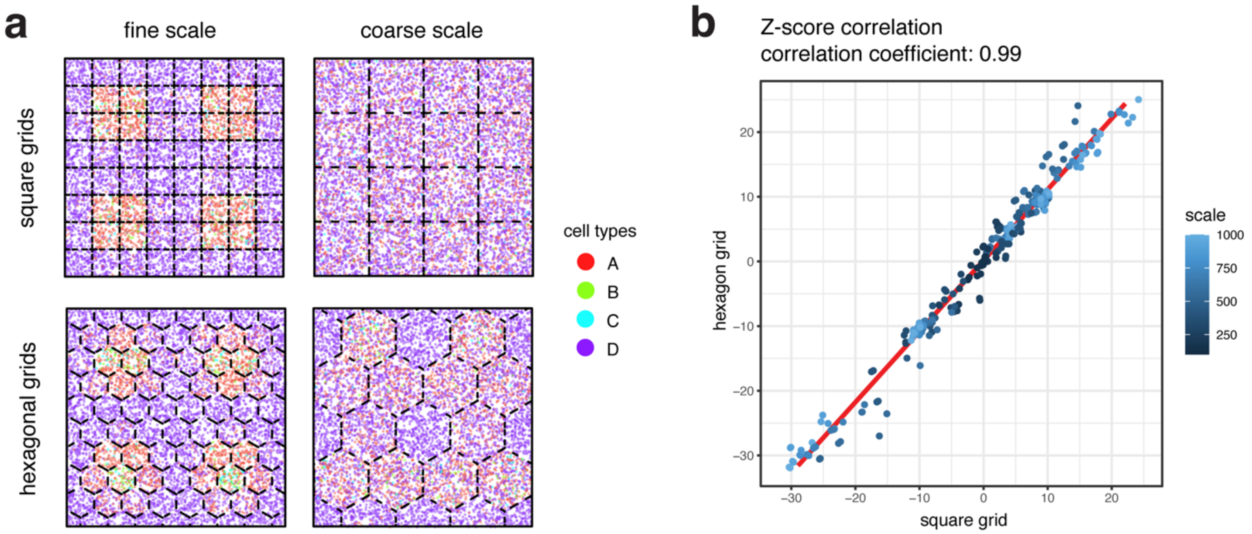

Supplementary Figure 9. Correlation between square and hexagonal grids. **a**. Visualization of the shuffled labels using the square and hexagonal grid tiles on simulated dataset for finer and coarser scales. **b**. Consistency of Z-scores and scales for the different grid tile shapes. The x-axis represents the Z-score obtained using the square grid tiles and the y-axis represents the Z-score obtained using the hexagon grid tiles. The color saturation represents the scale in which the value was obtained. The red line represents the line of best fit.

Supplementary Tables

Supplementary Table 1. Information on the MERFISH mouse brain samples. Description of the number of cells, unique cell types, and the tissue size in micrometers for each MERFISH mouse brain sample.

Location	Replicate	Number of Cells	Number of Cell Types	Tissue Size (µm)
1	1	78329	13	9036.87x6326.88
	2	88884	13	8060.09x9936.43
	3	84635	14	8504.56x8249.1
2	1	83546	14	8883.69x7113.93
	2	84171	14	8867.34x9316.67
	3	85957	14	9147.76x6980.63
3	1	70844	14	7058.34x7829.3
	2	83461	14	8952.54x6747.24
	3	74866	14	8952.54x6747.24
