# Supplementary material for: Characterizing cell-type spatial relationships across length scales in spatially resolved omics data
Source: Nat Commun. 2025 Jan 3;16:350. doi: 10.1038/s41467-024-55700-1 (PMC11699133; doi:10.1038/s41467-024-55700-1)
Supplement: Supplementary file 2 — Description of Additional Supplementary Files [file 41467_2024_55700_MOESM2_ESM.pdf]

Description of Additional Supplementary Files:

Supplementary Software 1: the CRAWDAD R Package
