# Supplementary material for: Characterizing cell-type spatial relationships across length scales in spatially resolved omics data
Source: Nat Commun. 2025 Jan 3;16:350. doi: 10.1038/s41467-024-55700-1 (PMC11699133; doi:10.1038/s41467-024-55700-1)
Supplement: Supplementary file 4 — Reporting Summary [file 41467_2024_55700_MOESM4_ESM.pdf]

nature portfolio

Corresponding author(s): Jean Fan

Last updated by author(s): Nov 4, 2024

Reporting Summary

Nature Portfolio wishes to improve the reproducibility of the work that we publish. This form provides structure for consistency and transparency in reporting. For further information on Nature Portfolio policies, see our Editorial Policies and the Editorial Policy Checklist.

Statistics

For all statistical analyses, confirm that the following items are present in the figure legend, table legend, main text, or Methods section.

n/a	Confirmed	
☐	☒	The exact sample size (*n*) for each experimental group/condition, given as a discrete number and unit of measurement
☐	☒	A statement on whether measurements were taken from distinct samples or whether the same sample was measured repeatedly
☐	☒	The statistical test(s) used AND whether they are one- or two-sided<br>*Only common tests should be described solely by name; describe more complex techniques in the Methods section.*
☒	☐	A description of all covariates tested
☐	☒	A description of any assumptions or corrections, such as tests of normality and adjustment for multiple comparisons
☐	☒	A full description of the statistical parameters including central tendency (e.g. means) or other basic estimates (e.g. regression coefficient) AND variation (e.g. standard deviation) or associated estimates of uncertainty (e.g. confidence intervals)
☐	☒	For null hypothesis testing, the test statistic (e.g. *F*, *t*, *r*) with confidence intervals, effect sizes, degrees of freedom and *P* value noted<br>*Give P values as exact values whenever suitable.*
☒	☐	For Bayesian analysis, information on the choice of priors and Markov chain Monte Carlo settings
☒	☐	For hierarchical and complex designs, identification of the appropriate level for tests and full reporting of outcomes
☒	☐	Estimates of effect sizes (e.g. Cohen's *d*, Pearson's *r*), indicating how they were calculated

Our web collection on statistics for biologists contains articles on many of the points above.

Software and code

Policy information about availability of computer code

Data collection	No data was collected. Only publicly available data was analyzed. The simulated datasets and the human spleen cell-type annotations created for this paper are available in CRAWDAD's Zenodo repository (https://doi.org/10.5281/zenodo.14004433).
Data analysis	The CRAWDAD software R package version 1.0.0 was used to analyze the data. The source code is publicly available at https://github.com/JEFworks-Lab/CRAWDAD (https://doi.org/10.5281/zenodo.14026290). The scripts used in the revision are available at https://github.com/rafaeldossantospeixoto/crawdad_revision_analysis (https://doi.org/10.5281/zenodo.14026360). We used spatstat (version 3.0-6) and Squidpy (version 1.2.3) packages.

For manuscripts utilizing custom algorithms or software that are central to the research but not yet described in published literature, software must be made available to editors and reviewers. We strongly encourage code deposition in a community repository (e.g. GitHub). See the Nature Portfolio guidelines for submitting code & software for further information.

Data

Policy information about availability of data

All manuscripts must include a data availability statement. This statement should provide the following information, where applicable:

- Accession codes, unique identifiers, or web links for publicly available datasets
- A description of any restrictions on data availability
- For clinical datasets or third party data, please ensure that the statement adheres to our policy

All data analyzed with CRAWDAD is publicly available. The simulated datasets are available in CRAWDAD’s Zenodo repository (https://doi.org/10.5281/

zenodo.14004433). The Slide-seqV2 mouse cerebellum dataset was obtained from the original publication (https://doi.org/10.1038/s41587-020-0739-1), with cell types previously annotated in RCTD (https://doi.org/10.1038/s41587-021-00830-w), available at the Broad Institute Single Cell Portal at https://singlecell.broadinstitute.org/single_cell/study/SCP948. The seqFISH mouse embryo data was obtained from the original publication (https://doi.org/10.1038/s41587-021-01006-2), available at https://doi.org/10.18129/B9.bioc.MouseGastrulationData. The Xenium human breast cancer dataset was obtained from the original publication (https://doi.org/10.1038/s41467-023-43458-x), available at the GEO database under accession code GSE243280 https://www.ncbi.nlm.nih.gov/geo/query/acc.cgi?acc=GSE243280. The MERFISH mouse brain datasets were obtained from the Vizgen Data Release V1.0. May 2021 (https://info.vizgen.com/mouse-brain-map), with cell types previously annotated in STalign (https://doi.org/10.1038/s41467-023-43915-7), available at https://doi.org/10.5281/zenodo.10724029. The CODEX human spleen samples were obtained from HuBMAP's data portal (https://doi.org/10.35079/HBM389.PKHL.936, https://doi.org/10.35079/HBM772.XXCD.697, https://doi.org/10.35079/HBM342.FSLD.938, https://doi.org/10.35079/HBM825.PBVN.284, https://doi.org/10.35079/HBM556.KSFB.592, https://doi.org/10.35079/HBM568.NGPL.345), with the cell type annotations performed in this paper available in CRAWDAD's Zenodo repository (https://doi.org/10.5281/zenodo.14004433).

Research involving human participants, their data, or biological material

Policy information about studies with human participants or human data. See also policy information about sex, gender (identity/presentation), and sexual orientation and race, ethnicity and racism.

Reporting on sex and gender	We did not perform sex- and gender-based analysis.
Reporting on race, ethnicity, or other socially relevant groupings	We did not use any socially constructed or socially relevant categorization variable.
Population characteristics	We did not use any socially constructed or socially relevant categorization variable.
Recruitment	No human research subjects were recruited for this study.
Ethics oversight	Not applicable.

Note that full information on the approval of the study protocol must also be provided in the manuscript.

Field-specific reporting

Please select the one below that is the best fit for your research. If you are not sure, read the appropriate sections before making your selection.

[x] Life sciences [ ] Behavioural & social sciences [ ] Ecological, evolutionary & environmental sciences

For a reference copy of the document with all sections, see nature.com/documents/nr-reporting-summary-flat.pdf

Life sciences study design

All studies must disclose on these points even when the disclosure is negative.

Sample size

The pre-processed subset of the seqFISH data contained 19416 cells with x-y coordinates and 22 cell-type annotations.

The pre-processed subset of the Slide-seq dataset contained 10,098 beads with x-y coordinates and 19 cell-type annotations previously predicted by RCTD.

The pre-processed Xenium dataset contained 162107 cells with x-y coordinates and 20 cell-type annotations.

The pre-processed MERFISH datasets contained 734693 cells with x-y coordinates and 14 cell-type annotations.

The pre-processed CODEX datasets of IDs HBM389.PKHL.936, HBM772.XXCD.697, HBM556.KSFB.592, HBM825.PBVN.284, HBM568.NGPL.345, and HBM342.FSLD.938 contained protein expression for 28 markers, x-y coordinates, and cell segmentation area measurements for 154,446, 150,311, 152,896, 130,584, 177,777, and 226,384 segmented cells, respectively.

Data exclusions

In the pre-processed subset of the Slide-seq dataset, poorly represented cell types defined as those being annotated in less than 20 beads (Choroid, Candelabrum, Ependymal, Globular, Macrophages) were not considered in the CRAWDAD cell type colocalization, resulting in 14 remaining cell types.

In the pre-processed MERFISH mouse brain datasets, cells that expressed less than three genes were removed. Additionally, subsets of the same cell type were merged into one.

In the pre-processed Xenium breast cancer dataset, cells that expressed less than three genes were removed.

Replication

We analyzed mouse brain samples from three distinct locations in three replicates, totaling nine datasets. The spatial relationships were consistent within the samples from the same brain location. The paper presents the figures for each sample's colocalization results.

We analyzed two spleen samples from three donors, totaling six different datasets. The spatial relationship of most cell-type pairs was consistent across samples, with some patient-specific or sample-specific relationships. The paper presents the figures for each sample's colocalization results.

Randomization	Randomization of samples is not relevant to this study because the spatial relationships found should reflect the biological sample analyzed.
Blinding	Blinding is not relevant to this study because the spatial relationships found should reflect the biological sample analyzed.

Reporting for specific materials, systems and methods

We require information from authors about some types of materials, experimental systems and methods used in many studies. Here, indicate whether each material, system or method listed is relevant to your study. If you are not sure if a list item applies to your research, read the appropriate section before selecting a response.

Materials & experimental systems

n/a	Involved in the study	
☒	☐	Antibodies
☒	☐	Eukaryotic cell lines
☒	☐	Palaeontology and archaeology
☒	☐	Animals and other organisms
☒	☐	Clinical data
☒	☐	Dual use research of concern
☒	☐	Plants

Methods

n/a	Involved in the study	
☒	☐	ChIP-seq
☒	☐	Flow cytometry
☒	☐	MRI-based neuroimaging

Plants

Seed stocks	*Report on the source of all seed stocks or other plant material used. If applicable, state the seed stock centre and catalogue number. If plant specimens were collected from the field, describe the collection location, date and sampling procedures.*
Novel plant genotypes	*Describe the methods by which all novel plant genotypes were produced. This includes those generated by transgenic approaches, gene editing, chemical/radiation-based mutagenesis and hybridization. For transgenic lines, describe the transformation method, the number of independent lines analyzed and the generation upon which experiments were performed. For gene-edited lines, describe the editor used, the endogenous sequence targeted for editing, the targeting guide RNA sequence (if applicable) and how the editor was applied.*
Authentication	*Describe any authentication procedures for each seed stock used or novel genotype generated. Describe any experiments used to assess the effect of a mutation and, where applicable, how potential secondary effects (e.g. second site T-DNA insertions, mosiacism, off-target gene editing) were examined.*
